# The Effectiveness of Mindfulness in the Treatment of Methamphetamine Addiction Symptoms: Does Neuroplasticity Play a Role?

**DOI:** 10.3390/brainsci14040320

**Published:** 2024-03-27

**Authors:** James Chmiel, Agnieszka Malinowska, Filip Rybakowski, Jerzy Leszek

**Affiliations:** 1 Institute of Neurofeedback and tDCS Poland, 70-393 Szczecin, Poland; 2 Institute of Psychology, University of Szczecin, 71-017 Szczecin, Poland; 3 Department and Clinic of Psychiatry, Poznan University of Medical Sciences, 61-701 Poznań, Poland; 4 Department and Clinic of Psychiatry, Wrocław Medical University, 54-235 Wrocław, Poland

**Keywords:** methamphetamine, mindfulness, addiction, methamphetamine use disorder

## Abstract

Introduction: Methamphetamine is a highly stimulating psychoactive drug that causes life-threatening addictions and affects millions of people around the world. Its effects on the brain are complex and include disturbances in the neurotransmitter systems and neurotoxicity. There are several known treatment methods, but their effectiveness is moderate. It must be emphasised that no drugs have been approved for treatment. For this reason, there is an urgent need to develop new, effective, and safe treatments for methamphetamine. One of the potential treatments is mindfulness meditation. In recent years, this technique has been researched extensively in the context of many neurological and psychiatric disorders. Methods: This review explores the use of mindfulness in the treatment of methamphetamine addiction. Searches were conducted in the PubMed/Medline, Research Gate, and Cochrane databases. Results: Ten studies were identified that used mindfulness-based interventions in the treatment of methamphetamine addiction. The results show that mindfulness is an effective form of reducing hunger, risk of relapses, stress indicators, depression, and aggression, alone or in combination with transcranial direct current stimulation (tDCS). Mindfulness also improved the cognitive function in addicts. The included studies used only behavioural measures. The potential mechanisms of mindfulness in addiction were explained, and it was proposed that it can induce neuroplasticity, alleviating the symptoms of addiction. Conclusions: Evidence from the studies suggest that mindfulness may be an effective treatment option for methamphetamine addiction, used alone or in combination with tDCS. However, further high-quality research is required to establish the role of this treatment option in this field. The use of neuroimaging and neurophysiological measures is fundamental to understand the mechanisms of mindfulness.

## 1. Introduction

Progress in medicine and neuroscience has enabled us to learn and understand the pathophysiological mechanisms of many neurological and psychiatric diseases. Detailed insights into these mechanisms allow us to develop new treatment methods. Among many psychiatric diseases, addictions play an important role. Numerous discoveries regarding addictions should, in principle, provide many potentially effective therapeutic interventions, however this is not the case. The development of new interventions does not keep up with the development and discoveries of new phenomena related to addictions. This problem particularly applies to behavioural interventions that were developed decades ago, when our understanding of addiction was less advanced. These types of interventions should not be ruled out. Their impact on neurological and psychiatric functioning is more complex than it may seem. This mechanistic complexity raises the hope that in some areas it will overlap with the mechanistic complexity of the pathophysiology of a given disorder and become an appropriate method of combating it.

### 1.1. Methamphetamine Use Disorder

The use of methamphetamine, a highly addictive central nervous system stimulant [1], poses a serious threat to public health due to its acute side effects, which include a substantial propensity for dependence [2], long-term neurotoxicity [3], and hyperthermia [4]. Methamphetamine is an illicitly manufactured recreational drug that is made from several chemicals found in over-the-counter drugs such as pseudoephedrine or ephedrine [5]. Methamphetamine can be ingested orally, injected, snorted, or smoked [6]. Methamphetamine has a lengthy 12 h half-life and, depending on how it is administered, the altered state of mind may persist for up to 12 h [7].

After cannabis, methamphetamine is the most-used illicit substance in the world [8]. The use of methamphetamine in the USA increased from 1.4 million in 2016 to 2.0 million in 2019, according to nationally representative data from the National Survey on Drug Use and Health (NSDUH). The prevalence of past-year use also increased from 0.5% in 2016 to 0.7% in 2019 [9]. Methamphetamine is used more often by men than women [10], and according to reports, the average yearly usage of methamphetamine among men over the age of 18 was 8.7 per 1000 individuals, while it was 4.7 per 1000 women [11].

Mechanistically, methamphetamine predominantly triggers the release of norepinephrine [12], serotonin [13], and dopamine [14]. Its ingress into the brain occurs via presynaptic neurons, where it primarily acts by reversing the vesicular monoamine transporter-2 (VMAT) [15]. This impedes the incorporation of neurotransmitters into vesicles, fostering the rapid efflux of intravascular monoamines and culminating in abnormally elevated cytosolic monoamine concentrations [3]. Consequently, overstimulation of monoaminergic pathways ensues in both the central and peripheral nervous systems [16], with higher doses provoking excitotoxicity via increased glutamate levels in the striatum [16]. Acting as a competitive antagonist on presynaptic DA receptors and a weak agonist on postsynaptic DA receptors, methamphetamine indirectly stimulates these receptors via dopamine release. Furthermore, methamphetamine inflicts neuronal damage within specific brain regions, alongside dopaminergic and serotonergic terminal degeneration [16].

The misuse of methamphetamine can have a variety of negative short- and long-term effects on one’s health. The short-term effects of methamphetamine are mainly related to increased dopamine release, which activates the brain’s reward system and induces euphoria [17], improves mental clarity [18], encourages “rush” [17], elevates mood [19], and reduces social and sexual inhibitions [20], all of which can result in substance dependency [21]. The reward system’s neuronal alterations brought on by this supraphysiologic dopamine release result in tolerance and drug-seeking behaviour [22,23]. Talkativeness [24], aggression [25], and restlessness [26] are also brought on by higher dosages. The short-term physiological effects of methamphetamine include elevated body temperature [27], mydriasis [28], tachycardia [29], hypertension [29], and appetite loss [30]. Psychotic symptoms like violent behaviour, paranoia, and hallucinations are likely to be among the longer-term effects [31]. People may display neurological and psychological symptoms like anxiety and depression [32]. Extreme itching, malnutrition, dental problems (also referred to as “meth mouth”), insomnia, and mood swings are some other potential long-term effects [33]. According to a meta-analysis, those who use methamphetamine may experience neuropsychological deficits that include problems with language, decision-making, information processing speed, and vasoconstriction skills [34]. Methamphetamine users have a higher prevalence of Parkinsonism [35]. According to reports, those who utilise methamphetamine are 75% more likely to get Parkinson’s disease than those who do not use methamphetamine [36]. It is important to remember that long-term methamphetamine usage has been linked to neurological and neurotoxic brain damage [37,38,39]. Methamphetamine also has detrimental effects on the peripheral organs. These include cardiovascular disorders, which are in second to accidental overdoses as the primary cause of death for users of methamphetamine [40].

The effects of methamphetamine use extend beyond health to include social and environmental impacts. Based on the data available for 2005, the RAND Corporation released the first national estimate of the economic cost of methamphetamine use in 2009. They calculated that the economic cost of methamphetamine use in the United States was roughly $23.4 billion. This figure included expenses for drug treatment, other medical expenses, the intangible cost of addiction and early death, lost productivity, costs related to crime and the criminal justice system, endangerment of children, and harms from production [41]. Methamphetamine users are more likely to live in rural areas and unstable housing, as well as have low incomes [42]. A higher likelihood of treatment non-adherence and missed appointments may result from a high frequency of co-occurring addiction and mental health issues [43].

There are several approaches to treating methamphetamine dependency. The first group are psychosocial interventions. Many individuals with concerns about their methamphetamine usage continue to seek twelve-step groups like Narcotics Anonymous as a typical method of intervention [44]. Studies most frequently focus on contingency management (CM) and/or cognitive-behavioural therapy (CBT). Even over very short treatment periods of two to four sessions, CBT treatment appears to be associated with reductions in methamphetamine usage and other favourable outcomes. According to CM research, levels of methamphetamine usage are significantly decreased when the technique is applied [45]. Repetitive transcranial magnetic stimulation (rTMS), exercise, residential rehabilitation-based therapies, and the matrix model all show promise in helping the participants quit using methamphetamine and lessen their cravings [46]. To date, there are no approved drugs to treat methamphetamine dependency [47]. Clinical trials with aripiprazole, SSRIs, ondansetron, mirtazapine, and GABA drugs (gabapentin, baclofen, and vigabatrin) have demonstrated no significant effectiveness [48].

Due to the insufficient number of approaches to treating methamphetamine dependency, it is necessary to develop new and effective methods that are as non-invasive and safe as possible. One such method may be mindfulness.

### 1.2. Mindfulness

Mindfulness meditation is defined as awareness of the events taking place, while refraining from judging them [49]. This concept has its roots in the Buddhist, Islamic, Christian, and Judaic religions [50,51,52]. A fundamental aspect of mindfulness meditation is non-interference in cognitive processes. Therefore, mindfulness involves being aware of thoughts, emotions, and external events, whilst refraining from judgement and interference. Instead, acceptance and kindness is maintained. When engaging in mindfulness practice, the primary objective is to remain present in the moment, abstaining from dwelling on past occurrences or envisioning future scenarios. According to the field of cognitive-behavioural psychotherapy, emerging thoughts generate affective states and the subject merges with their thoughts [53]. Mindfulness brings individuals towards the process of defusion, which involves noticing a thought and remaining with it without becoming attached to its content [54,55]. Mindfulness may be informally integrated into daily life by consciously experiencing situations, disconnecting from their evaluations, and attentively observing emerging internal states [56]. Another formal form of mindfulness is meditation [8]. Based on meditation, cognitive-behavioural psychotherapy offers therapeutic protocols using mindfulness practice, e.g., mindfulness-based stress reduction (MBSR), which supports the ability to recognise one’s thoughts without judging them. This can lead to the regulation of the emotions and behaviours. [57]. Basic MBSR, as well as its variants (mindfulness-based interventions (MBI), mindfulness-based cognitive therapy (MBCT), and mindful-self compassion (MSC) [58]), consist of 8–10 weekly 2.5 h classes with 10–40 participants, a full-day retreat, and roughly 45 min of formal and informal practice each day [59]. During the basic sessions, participants learn how to practice meditation and breathing techniques, catch distracting stimuli, observe emerging thoughts or emotions, and accept thoughts as they appear.

Mindfulness is extensively researched in the context of treating a multitude of disorders, including neurological and psychiatric ones. For example, there is evidence that mindfulness is effective in alleviating symptoms of ADHD [60], autism [61], insomnia [62], Parkinson’s disease [63], and chronic pain [64]. Meta-analyses also provide information on the positive relationships of mindfulness with working memory, executive functions, inhibitory control, and cognitive flexibility [65,66]. Mindfulness practices also result in reduced arousal in the resting network, which reduces the number of wandering thoughts [67]. It also has a positive relationship with the occurrence of anxiety and depressive disorders [68]. The practice of mindfulness has a positive impact on maintaining the body’s homeostasis [69], emotional stability [70], increasing empathy [71], reducing anxiety, and regulating the functioning of the sympathetic nervous system [72]. Mindfulness is a safe method, and the overall rate of adverse events is 8.3% [73].

To date, several reviews have been published examining the effectiveness of mindfulness in the treatment of addictions, including cocaine, alcohol, and tobacco. However, few studies on methamphetamine dependency have been included. Moreover, the potential of mindfulness in the context of inducing neuroplasticity (which may contribute to reducing the symptoms of dependency) was not addressed. The objective of this review is to address this deficiency.

## 2. Methods

### 2.1. Data Sources and Search Strategy

For this review, J. Ch., A. M., F. R., and J. L. performed an independent online search using predefined criteria. The following combined keywords were used: “mindfulness” AND “methamphetamine” OR “meth” OR “stimulant” OR “amphetamine.” Publications in the PubMed/Medline, Research Gate, and Cochrane databases, with an access date of January 2024 and publication dates ranging from January 2000 to January 2024, were considered.

### 2.2. Study Selection Criteria

Eligibility criteria included clinical trials conducted in English, published from the years 2000 to 2024. Considered studies investigated the effects of mindfulness on methamphetamine dependency as a primary or secondary outcome. The exclusion criteria encompassed articles that were not published in English, as well as literature reviews.

### 2.3. Screening Process

The screening process was conducted in multiple stages to ensure the inclusion of relevant studies and the exclusion of those that did not meet the predefined criteria. The initial screening involved a thorough examination of titles and abstracts by the reviewers (J. Ch., A. M., F. R., and J. L.) independently.

#### 2.3.1. Title and Abstract Screening

To find studies that would fit the inclusion requirements, each reviewer evaluated the titles and abstracts of the records they were able to obtain independently. Relevance to mindfulness and its impact on methamphetamine-addicted patients were the main screening criteria.

#### 2.3.2. Full-Text Assessment

After screening for titles and abstracts, the chosen papers were subjected to a thorough full-text evaluation. Reviewers stressed the inclusion of clinical trials conducted in English that were published between January 2000 and January 2024. Articles were examined in their entirety to ascertain whether they met the specific eligibility requirements.

## 3. Results

**Figure 1:**
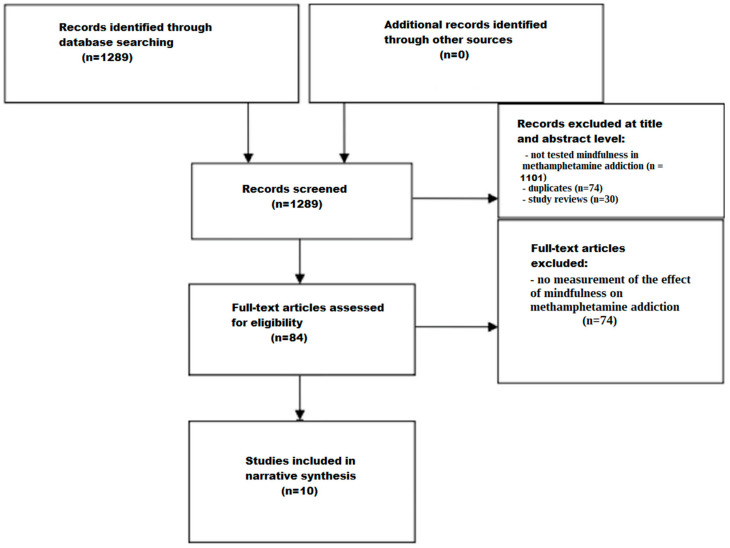
Flow chart depicting different phases of systematic review.

The screening process is illustrated in a flow chart (Figure 1). Through the search strategies carried out in the databases, 1289 studies were identified. A total of 1205 studies were excluded based on the evaluation of their titles and abstracts, due to not testing mindfulness in methamphetamine dependency (*n* = 1101), due to removal of duplicates (*n* = 74), and due to removal of study reviews (*n* = 30). Afterwards, 84 studies were identified and underwent a comprehensive full-text assessment. Of these, 74 studies were excluded because they did not measure the effect of mindfulness on methamphetamine dependency. After a full reading of the texts, 10 articles were deemed eligible for inclusion.

### 3.1. Summary of Included Studies

The studies included in the review are presented in Table 1. In the quasi-experimental study conducted by Hosseini et al. [74], they investigated how a MBRP therapy affects the desire for drugs and the ability to manage emotions among individuals in a therapeutic community center. The study involved 40 participants, with half assigned to the MBRP group and the other half assigned to the control group. The MBRP group attended eight 90 min sessions of therapy, one per week, while the control group did not receive any treatment and remained on the waiting list. They used the Drug Craving Questionnaire to assess drug cravings and the Emotion Regulation Questionnaire to gauge emotional well-being.

Maneesang et al. [75] conducted a study to investigate how effective a program called mindfulness-based therapy and counseling (MBTC) is in treating methamphetamine use disorder. They studied 70 individuals who were already receiving treatment for methamphetamine dependency. The participants were split into two groups: a control group that followed the regular treatment program and an experimental group that combined MBTC with the regular program at a treatment center. The researchers used the Methamphetamine Craving Questionnaire, Urine Colour Immunochromatographic Assay, and the Mini-International Neuropsychiatric Interview to evaluate the impact of the treatment. The effects on depression, stress, and relapses were also measured. The MBTC program involved weekly mindfulness practice sessions lasting 90–120 min over a period of 8 weeks. The participants were evaluated before and after the treatment, and during follow-up visits at 2, 4, 8, 12, and 24 weeks.

Zhai et al. [76] conducted a study to investigate how MBRP impacts individuals undergoing compulsory isolation detoxification treatment for methamphetamine dependence. Forty-one patients with methamphetamine dependence participated in the study. The participants were randomly assigned to either the MBRP group (consisting of 21 individuals) or the control group (consisting of 20 individuals). The MBRP group attended 16 sessions of 2 h MBRP interventions, twice a week over 8 weeks. The researchers conducted assessments at the beginning, as well as the fourth and eighth week into the intervention. To assess the effects of the treatment, researchers used the Chinese version of the Beck Depression Inventory (BDI) to measure depression levels, the Chinese version of the State-Trait Anxiety Inventory (STAI) to gauge anxiety, and the Visual Analog Scale (VAS) to measure the intensity of drug craving.

Dsouza et al. [77] conducted a study to explore how MBCT impacts individuals who use methamphetamine. They had 30 participants who were randomly divided into the experimental and control groups, with 15 people in each group. The researchers used the Holmes and Rahe stress questionnaire and the Somoza Craving Questionnaire to assess the participants’ stress levels and cravings, respectively. Additionally, they analysed the participants’ urine to measure cortisol levels. The experimental group attended eight 60 min sessions of mindfulness therapy, whilst the control group did not receive any treatment.

The purpose of the Hamidi and Kheiran study [78] was to find out how well educational treatments based on MBRP work to lessen high-risk behaviours in methamphetamine-dependent individuals, such as aggression and cravings. Based on DSM-5 criteria, 30 male and female patients were diagnosed with methamphetamine dependence. They were then randomly assigned to two groups, each consisting of 15 subjects—an experimental group and a control group. The Craving Beliefs Questionnaire (CBQ) and the Aggression Questionnaire by Eysenck and Wilson were finished by the two groups. After completing the intervention sessions, the variables in the post-test stage were remeasured in two groups. The experimental group had eight 1.5 h sessions of preventive training from MBRP.

Alizadehgoradel et al. [79] conducted a study to explore how practicing mindfulness can enhance the executive functions of a group of adolescents dealing with methamphetamine use disorders, as evaluated through neuroscientific tools. They included 40 adolescents who were randomly assigned to either the experimental group (consisting of 20 individuals) or the control group (also consisting of 20 individuals). The participants were assessed at three different time points: before the intervention, immediately after the intervention, and one-month post-treatment. The researchers administered the mindfulness-based substance abuse treatment (MBSAT) over the course of 12 sessions, with 2 sessions lasting 50–60 min per week. To evaluate the impact of the treatment, they utilized the Go/No-Go test to assess response inhibition, the Balloon Analogue Risk Task (BART) to evaluate risky behaviour and decision-making, the Wisconsin Card Sorting Task (WCST) to measure cognitive flexibility, and the N-back task to assess working memory.

In the research of Shareh et al. [80], 30 methamphetamine-dependent individuals were sorted at random into two experimental (*n* = 15) and control (*n* = 15) groups following the detoxification phase. The experimental group participated in nine one-hour mindfulness sessions (twice a week) of training in the MBRP paradigm, while the control group participated in the center’s regular group therapy program. Before and after treatment, the two groups performed the Beck Depression Inventory, Second Edition (BDI-II) in order to gauge depression, as well as the Relapse Prediction Scale (RPS) to gauge craving.

In the study by Alizadehgoradel et al. [81], the aim was to investigate the use of MBSAT combined with transcranial direct current stimulation (tDCS) in 80 young adults addicted to methamphetamine. There were four groups of patients: tDCS alone (*n* = 20), mindfulness alone (*n* = 20), a combination of mindfulness and tDCS (*n* = 20), and a sham group (*n* = 20). The N-back task was used to measure working memory, the WCST was used to measure cognitive flexibility, the BART was used to measure risky behaviour and decision-making, the Go/No-Go task was used to measure inhibitory control, and the Desires for Drug Questionnaire (DDQ) was used to measure craving.

The study of Alizadehgoradel et al. [82] looked at how mindfulness and tDCS affected methamphetamine-addicted adolescents’ Attentional Bias (AB) toward drug-related stimuli and cravings. A total of 48 adolescents who were addicted to methamphetamine were divided into three research groups at random: a sham group (12 sessions), a combination mindfulness + tDCS group (12 sessions mindfulness + 12 sessions tDCS), and a mindfulness group (12 sessions conducted twice per week). A 50 min mindfulness-based substance addiction treatment (MBSAT) intervention was used for 12 sessions, twice a week. Measures of AB and craving were taken prior to the intervention, following the 12 sessions, and at the one-month follow-up. The dot-probe task was used to measure AB and the DDQ was used to measure craving.

In the Alizadehgoradel study [83], 80 adolescent boys with early abstinent methamphetamine dependency were included. The research groups—tDCS group (*n* = 20), mindfulness group (*n* = 20), combined mindfulness-tDCS group (*n* = 20), and sham group (*n* = 20)—were allocated to participants at random. Mindfulness meditation was based on the MBSAT method. The 12-session mindfulness regimen (two sessions per week) was followed by the MBSAT group. The duration of each mindfulness treatment session was 40–50 min. Negative emotions were measured using the Depression, Anxiety, and Stress Scales (DASS-21), while craving was measured using the DDQ. 

### 3.2. Effect of Mindfulness on Different Outcomes in Methamphetamine Dependency

Regarding craving, mindfulness improved results in a total of eight studies [74,75,77,78,80,81,82,83] and did not improve the results in one [76]. Citing more detailed results, in [75], at the six-month check, the group that had received the experimental treatment showed a noticeable decrease in cravings compared to the other group. Specifically, their craving was significantly lower by 7.89 points, with a 95% confidence interval ranging from −15.47 to −0.32. In [78], the MBRP intervention was found to decrease cravings. Additionally, the impact of the intervention on reducing cravings was estimated to be around 27%, as indicated by the Eta coefficients obtained during the analysis. In [80], the one-way analysis of the covariance results demonstrated a significant difference favouring the greater efficiency of mindfulness-based therapy in the post-test variables of likelihood of drug use (F = 10.2, *p* < 0.01) and substance craving (F = 35.8, *p* < 0.01). In [81,82,83], craving scores improved with the combination of MBSAT and tDCS and MBSAT alone, but the improvement was greater with the combined intervention.

Regarding depression, mindfulness improved these results in three studies [75,80,83] and did not improve the results in one [76]. In [75], the experimental group had significantly lower depression scores during the six-month follow-up period (−2.95, 95%CI = −5.31, −0.6). In [80], the one-way analysis of the covariance results demonstrated a significant difference in favour of mindfulness-based therapy’s increased effectiveness in the depressive variables (F = 15.49, *p* < 0.01) at the post-test. In [83], depression scores improved after mindfulness and a combined intervention, but the improvement was greater after the combined intervention.

Regarding stress and anxiety, in [75], the experimental group had significantly lower stress scores during the 6-month follow-up period (−7.44, 95%CI = −12.21, −2.67). The study [76] showed no effect of MBRP on anxiety scores. In [77], mindfulness reduced the stress scores. In [83], both MBSAT alone and the combination of tDCS AND MBSAT improved anxiety and stress scores, while the improvement was greater after the combined intervention.

Regarding impact on relapses, [75] found that within the MBTC group, the rate of people experiencing a methamphetamine relapse was notably lower (5 out of 35,14.3%) compared to the control group (16 out of 35,45.7%).

Regarding effect on cortisol levels, mindfulness reduced the cortisol levels in [77].

Regarding emotion regulation, in [74], MBPR improved emotion regulation.

Regarding impact on aggression, [78] found that the MBRP intervention had an impact on reducing aggression. Additionally, the effect of the intervention on decreasing aggression was estimated to be approximately 53%, as suggested by the Eta coefficients obtained during the analysis.

Regarding the effect on response inhibition, two studies showed a positive effect. In [79], MBSAT improved response inhibition. The study [81] showed an increase and decrease of Accuracy Go and Accuracy No-Go, respectively, after the combined tDCS + MBSAT intervention but not after MBSAT alone.

Regarding the effect on working memory, two studies showed a positive effect. In [79], MBSAT improved working memory. In [81], MBSAT and the combination of tDCS and MBSAT improved accuracy and response time in a WM task, but the improvement was greater after the combined intervention.

Regarding impact on cognitive flexibility, two studies showed a positive effect. In [79], MBSAT improved cognitive flexibility. The study [81] showed a significant decrease and increase of WCST persistent errors and completed categories after MBSAT and the combination of tDCS and MBSAT, but the improvement was greater after the combined intervention.

Regarding the effect on risky behaviour and decision-making, two studies showed a positive effect. In [79], MBSAT improved risky behaviour and decision-making. The study [81] showed a significant decrease of BART adjusted value and max pumping, respectively, after MBSAT and combined tDCS and MBSAT, but the improvement was greater after the combined intervention.

Regarding the effect on attentional bias (AB), ref. [82] showed improvement in AB after MBSAT and the combination of tDCS and MBSAT, but the improvement was greater after the combined intervention.

## 4. Discussion

The examination of several studies highlights the diverse impacts of various mindfulness-based interventions on individuals dealing with substance abuse disorders, specifically methamphetamine dependency. The studies employed different methodologies and tools to measure the effects on various psychological and cognitive factors.

Regarding the impact on drug cravings, the results are generally positive. While the MBRP therapy in the Hosseini et al. [74] study appeared to alleviate drug cravings, the study by Maneesang et al. [75] demonstrated a significant decrease in craving levels for the experimental group at the six-month follow-up. Zhai et al. [76] did not find any significant effect of MBRP on craving. The study conducted by Dsouza et al. [77] suggested that mindfulness reduced craving scores, indicating a potential benefit in managing substance cravings. The intervention in [78] was found to decrease cravings, with an estimated impact of around 27%. Additionally, the Shareh et al. [80] study results demonstrated a significant reduction of likelihood of drug use and substance craving. Three studies [81,82,83] testing the effects of mindfulness and a combined mindfulness intervention with tDCS also found a beneficial effect of meditation on craving, but the combination of mindfulness and tDCS proved to be more effective.

When examining the effects on mental health factors, the studies by Maneesang et al. [75], Shareh et al. [80], and Alizadehgoradel [83] demonstrated that the experimental group had notably lower depression scores during the six-month follow-up and immediately after the intervention. Furthermore, ref. [75] indicated a significant reduction in stress levels for the experimental group. However, the research by Zhai et al. [76] did not find any notable effects of MBRP on depression or anxiety levels. Moreover, the study by Dsouza et al. [77] suggested a decrease in cortisol levels, indicating potential stress reduction as a result of mindfulness therapy. Alizadehgoradel study [83] showed that the combination of mindfulness and tDCS is more effective in reducing stress and anxiety than mindfulness alone (although it also lowered these results).

Considering the effect on relapses, Maneesang et al. [75] found that the rate of individuals experiencing methamphetamine relapses was notably lower within the group receiving mindfulness-based therapy.

Exploring the cognitive impacts, Alizadehgoradel et al. [79] demonstrated that mindfulness-based interventions can lead to improvements in executive functions, including response inhibition, working memory, cognitive flexibility, and risky decision-making behaviour. Similar effects were demonstrated in [81], where mindfulness improved the results, but the combination of mindfulness with tDCS was more effective in reducing risky decision making and improving many cognitive functions. In [82], the combination of interventions also improved attentional bias scores.

Furthermore, the study by Hamidi and Kheiran [78] indicated that educational interventions in MBRP can effectively reduce high-risk behaviours. The study suggested a reduction in aggression by approximately 53% following the intervention, emphasising the potential of mindfulness-based approaches in managing aggressive behaviours.

Overall, the findings underscore the promising role of mindfulness-based interventions in improving various psychological, cognitive, and behavioural aspects associated with methamphetamine dependence and suggest their potential as valuable components in comprehensive treatment programs for methamphetamine abuse disorders. Moreover, the combination of mindfulness with transcranial direct current stimulation shows even greater effectiveness in reducing the symptoms of methamphetamine dependency and improving cognitive functions. This suggests the therapeutic potential of combining both techniques against the condition, however, further research is required to explore the synergistic effect of tDCS and mindfulness.

## 5. Potential Mechanisms of Mindfulness in the Treatment of Methamphetamine Dependency

Little is known about how mindfulness works in methamphetamine dependency. This is because the included studies did not use any measures that examined the mechanisms. The following subsections discuss potential mechanisms, but it is important to emphasise that these are speculations based on the research of other addictions and disorders.

### 5.1. Impact on Craving

To measure the effects of mindfulness on cognitive and neuronal regulation of craving in addiction, neuroimaging methodologies such as functional magnetic resonance imaging (fMRI) can be employed. Studies have shown that mindfulness can modulate the neural circuitry associated with craving responses, indicating a potential for clinical significance [84]. Real-time fMRI provides a dynamic approach for evaluating the mindfulness-centered regulation of craving-related neural circuitry, offering direct measurements and feedback to guide mindfulness efforts. Mindfulness-based interventions (MBIs) offer a promising avenue for addressing cravings in methamphetamine dependency by leveraging both bottom-up and top-down processes [85]. These interventions operate on multiple fronts, targeting reactivity to conditioned cues and negative affective states while also enhancing executive control circuits [85]. The prefrontal cortex, particularly the dorsolateral prefrontal cortex (DLPFC), plays a crucial role in the top-down regulation of substance craving [86]. Studies suggest that mindfulness meditation can lead to structural changes in the DLPFC, potentially reinforcing the regulatory mechanisms needed for addiction recovery [87]. Additionally, increased gray matter volume in the right orbitofrontal cortex (OFC) among experienced meditators may contribute to improved emotional regulation [88].

Mindfulness training is demonstrating efficacy in reducing subjective craving and physiological indices of cue-reactivity [89]. It diminishes attentional bias towards drug cues [90] and decreases craving responses during cue-exposure protocols [91]. By decoupling negative emotions from craving, MBIs disrupt the typical association between the negative affect and substance use, potentially interrupting the cycle of addiction [89]. Mindfulness-oriented recovery enhancement (MORE) and MBRP have been particularly effective in reducing the association between mood states, craving, and substance use [85]. Various studies such as those on smoking cessation trials and mindfulness training for smokers demonstrate the effectiveness of MBIs in reducing striatal responses to drug cues [92]. The impact extends to clinical trials, where mindfulness-based addiction treatment shows the meditation effects on smoking abstinence through decreased craving [93].

MBIs contribute to therapeutic effects by heightening awareness of implicit craving responses [94]. Conscious craving, as proposed by Tiffany [95], is addressed by MBIs, making individuals more conscious of their appetitive drive to use substances. This increased awareness, achieved through interoceptive awareness and increased anterior insula activity during mindfulness meditation [68,96], plays a crucial role in preventing relapse, particularly in high-risk situations [94], suggesting improved access to internal cues.

Craving, characterised by subjective wanting and heightened incentive salience, is a critical driver in compulsive drug seeking and relapse [97]. Neural markers of incentive salience, such as activity in the subgenual ACC and ventral striatum, are significantly modified by MBIs [98,99].

### 5.2. Impact on Executive Functions

Executive functions, vital for reducing drug use and sustaining abstinence, are subject to enhancement through the reinforcement of top-down cognitive control by MBIs [89]. A quasi-experimental study focused on polysubstance use revealed substantial improvements in executive functioning, encompassing working memory, selective attention/response inhibition, and decision-making skills following mindfulness training [100]. The positive impact was replicated in a subsequent pilot RCT, emphasising the robustness of combined goal management and mindfulness training in both controlled laboratory settings and real-world decision-making scenarios [101]. The clinical realm echoes these findings, with mindfulness-based addiction treatment significantly enhancing smoking abstinence by alleviating concentration difficulties [93]. Exploring the neural underpinnings next, RCT exhibited reductions in smoking alongside heightened resting state activity in the anterior cingulate cortex (ACC) and the medial prefrontal cortex (mPFC) after just two weeks of mindfulness training [102]. This increased prefrontal activation aligns with the potential of mindfulness to disrupt automatic addictive responses [89].

A substantial body of evidence coalesces to underscore the efficacy of mindfulness training in fortifying inhibitory control capacity [66]. This extends to reduced self-reported impulsivity and enhanced performance on tasks measuring inhibitory control, such as the Go/No-Go task and the two-choice impulsivity paradigm [103]. Long-term mindfulness meditation training not only refines response inhibition but also exhibits sustained effects, underscoring the enduring impact of these practices [104]. Impressively, even brief mindfulness meditation interventions demonstrate the capacity to restore inhibitory control following self-control depletion [105]. The applicability extends to populations grappling with attention deficit/hyperactivity disorder [106] and addictive behaviours [107], where mindfulness training positively influences event-related potentials during Go/No-Go task performance. Moreover, in the realm of opioid addiction, an eight-week MORE program demonstrated enhanced inhibitory control during negative affective interference [108]. This aligns with neuroimaging evidence, where MBI participants showcased reduced errors on a response inhibition task alongside heightened dorsolateral prefrontal cortex (PFC) responses [109]. The impact extends to increased functional connectivity in circuits mediating intentional inhibition, underscoring the comprehensive influence of MBIs on the inhibitory control networks’ white matter integrity and functional connectivity [110,111].

### 5.3. Impact on Stress Reactivity

The established connection between stress and methamphetamine dependency [112,113] underscores the potential impact of MBIs in mitigating addictive behaviours by modulating stress reactivity and aiding stress recovery. Numerous studies employing heart rate variability (HRV) as an indicator of physiological reactivity and stress recovery reveal the positive effects of MBIs [114,115]. MBRP, for instance, demonstrated significantly greater increases in both tonic and phasic HRV responses to stress in individuals undergoing substance use disorder treatment [116]. Similarly, mindfulness training for those with alcohol and/or cocaine use disorders exhibited an attenuated sympathovagal HRV ratio during stress exposure [117]. In the context of nicotine-deprived smokers, brief mindfulness training correlated with higher HRV during stress exposure, highlighting the potential universality of these effects across various substances of abuse [118]. Additionally, MORE showed significant HRV recovery from stress-primed alcohol cues, coupled with reductions in cue-induced distress, providing further evidence of the stress-regulatory effects of MBIs [119]. Neuroimaging studies compliment these findings, highlighting the attenuation of amygdala and insula activation in response to stressful stimuli following mindfulness training [120].

Mindfulness training may not only influence emotional regulation, but facilitate the neurocognitive regulation of stress on the autonomic nervous system as well [94]. As individuals develop dispositional mindfulness through MBIs, they might enhance prefrontal cortical modulation of the sympathetic “fight-or-flight” response via parasympathetic nervous system activation, leading to increased HRV and heart-rate deceleration during stress or exposure to addictive cues [94]. This suggests that increased dispositional mindfulness might reflect greater neurovisceral integration and flexibility in the central autonomic network [121], enabling better contextual engagement and subsequent disengagement of neurocognitive resources in response to stress and drug-related stimuli [94]. The impact of mindfulness on neural function in key structures involved in central autonomic regulation, such as the dorsolateral prefrontal cortex (dlPFC) [122] and anterior cingulate cortex (ACC) [123], aligns with the proposed hypothesis. Notably, MBIs surpass relaxation therapy in increasing parasympathetically mediated HRV and decreasing stress indices, indicating their potential to improve substance cue-reactivity.

### 5.4. Impact on Reward System

Garland’s concept of the restructuring reward hypothesis lays the groundwork for understanding how MBIs may counteract the allostatic effects of addiction on reward neurocircuitry [124]. By enhancing functional connectivity between top-down and bottom-up brain circuitry, MBIs aim to reverse the allostatic process that shifts the valuation of natural rewards to drug-related rewards [125]. Mindfulness training, albeit not explicitly designed for this purpose, might enhance the pleasure derived from perceptual experiences, analogous to sensate-focus techniques [89]. This application, termed “savoring,” involves mindful attention to and appreciation of natural rewards, fostering positive effects and pleasure [126]. Studies supporting this hypothesis indicate that MBIs such as MORE can increase positive emotional responses and enhance reward processing, thereby reducing cravings and addictive behaviour [89].

MBIs have demonstrated their capacity to amplify the hedonic processing of natural rewards [94]. Studies show that MORE increases cardiac-autonomic and electrocortical responses to natural reward stimuli, leading to decreases in opioid craving [94]. Preliminary functional magnetic resonance imaging evidence reveals that MORE can induce pre-to-post treatment reductions in the ventral striatal responses to drug cues while increasing activation in reward-related brain regions during the savouring of natural rewards [92]. This suggests that MBIs may effectively rewire the reward circuitry, steering individuals away from drug-related stimuli and towards positive and natural rewards.

Repeated drug use often results in neural sensitisation, leading to heightened responses to drug-related stimuli and diminished responses to intrinsic rewards [92]. Through practices like savouring, MBIs teach individuals to mindfully attend to and appreciate pleasurable objects and experiences, potentially countering the allostatic effects of addiction on the reward neurocircuitry. Savouring, a potent positive emotion regulation strategy, involves sustained attention to the sensory features of the rewarding stimuli, simultaneously promoting metacognitive awareness of pleasant somatic and affective responses [94]. This intentional upregulation of positive emotions may offset negative affective states triggering addictive responses and restore the salience of intrinsically rewarding stimuli. Evidence suggests that mindfulness training can increase the reward experience and positive emotion, offering a promising avenue for addressing reward deficiency syndrome inherent in addiction [127,128].

Attention to positive information is a key component through which mindfulness ameliorates addiction, aligning with the restructuring reward hypothesis [94]. MBIs may facilitate a shift in the relative salience of drug and natural rewards, enhancing the pleasure and meaning derived from the latter. This shift occurs through techniques aimed at downregulating the exaggerated valuation of drug-related rewards while concurrently upregulating the perceived value of natural rewards [129]. Savouring, as a form of selective attention to positive experiences, is thought to overcome the “hedonic treadmill effect,” sustaining the pleasure derived from rewarding objects or events [129]. The generalisability of a mindfulness-induced positive affect across various contexts underlines the potential of MBIs in treating reward-related pathology and anhedonia associated with addiction [129].

### 5.5. Impact on Emotion Regulation

Emotion regulation, also known as affect monitoring and control, describes the mechanisms by which people choose which emotions to feel, when to feel them, and how to express and experience them [130]. It is a dynamic process and influences the onset, intensity, and expression of emotions [131]. The anterior cingulate cortex (ACC) and the medial prefrontal cortex (mPFC), two of the circuits of the brain, are critical for emotional regulation [132,133]. The possible influence of mindfulness meditation on emotion regulation has drawn attention from the clinical and scientific domains. A range of approaches have been utilised to examine the impact of mindfulness meditation on emotion regulation, such as self-report assessments, physiological measures, and neuroimaging analysis [134]. Empirical findings consistently demonstrate that implicit and explicit processes are combined in mindfulness-based emotion regulation [135]. Those practicing mindfulness have been shown to experience favourable changes in their emotional states, such as increased happy feelings and decreased negative emotions [49,136]. These results highlight the capacity of mindfulness meditation to modify the brain substrates linked to emotional regulation in addition to its effect on the subjective experience of emotions.

Emotion regulation in methamphetamine dependency is dysfunctional [137]. According to Payer et al. [138], an inverse correlation between aggressiveness committed by methamphetamine users and less amygdala activation during emotion regulation exists. This suggests that those who exhibit less violent behaviour are those who have reduced amygdala activation during regulation. The amygdala is important for several behavioural constructs related to the regulation of emotions in this population, as evidenced by another study that found resting state functional connectivity of the amygdala with the hippocampus in methamphetamine users is positively related to emotion dysregulation and trait anxiety and negatively related to self-compassion and dispositional mindfulness [139].

The neurobiological underpinnings of addiction involve a deficit in the self-control network, prominently featuring the anterior cingulate cortex (ACC) and the medial prefrontal cortex (mPFC) [140]. Integrative body-mind training (IBMT), a form of mindfulness meditation, has become a good tool for addressing this addiction-related issue. IBMT emphasises an attitude of acceptance and openness to both internal and external experiences through the methodical training of attention and self-control [140]. Following IBMT, studies have repeatedly shown that both the central and autonomic nervous systems experience quick and beneficial changes. These changes include lower levels of stress hormones, enhanced pleasant mood states, and structural alterations in the brain [141,142]. After brief IBMT sessions, the ACC and its neighbouring mPFC exhibit enhanced activity. This finding raises the possibility that mindfulness could improve emotion regulation in the context of addiction [143]. The central claim is that mindfulness meditation, like IBMT, can strengthen emotion regulation and thus increase self-control. Given that emotional dysregulation and decreased self-control are hallmarks of addiction symptoms, the ACC/mPFC’s role in emotional regulation becomes crucial to intervention attempts. Mindfulness meditation presents a viable option for the prevention and treatment of addiction by increasing activity in certain brain regions. A study that randomly assigned U.S. undergraduates to relaxation or IBMT groups investigated anatomical changes in white matter efficiency using diffusion tensor imaging as the experiment expanded to longer-term IBMT practice [111]. The findings showed that fractional anisotropy (FA) in the corona radiata, a white-matter tract that connects the ACC to other regions, increased after approximately 10 h of IBMT over the course of four weeks. This implied increased white matter efficiency, which might have an impact on emotion regulation and self-control skills. A longitudinal study that looked at radial diffusivity (RD) and axial diffusivity (AD) indices throughout two–four weeks of mindfulness meditation practice helped to further understand the dynamics of white matter alteration [110]. After four weeks of IBMT, the results showed increases in FA and decreases in both RD and AD. A complex pattern involving modifications to myelin and other axonal components around the ACC (a critical area in the brain network linked to self-control) suggested enhanced white matter efficiency. Understanding how mindfulness affects the brain’s reaction to emotions has been made possible by neuroimaging studies, which frequently use emotional cues. When in a mindful state, novice meditators often show decreased amygdala activity during emotional stimuli, suggesting a possible reduction in emotional arousal [68,144]. This reduced amygdala response is consistent with the theory that mindfulness enhances prefrontal cognitive control, which in turn reduces activity in areas of the brain involved in processing emotions [145].

## 6. Can Mindfulness Induce Neuroplasticity in Addictions?

Neuroplasticity, which is sometimes referred to as neural plasticity or brain plasticity, is a process in which the brain adapts its structure and functions. It is the nervous system’s capacity to rearrange its connections, functions, or structure in response to external or internal inputs [146]. Structural and functional plasticity are the two main categories into which it falls. The remodeling of spines, dendrites, and/or axons causes the synaptic region to expand or contract, which is referred to as structural plasticity [147]. Reorganising synaptic components and receptors, controlling neurotransmission, and adjusting the strength or efficiency of synaptic transmission are all examples of functional plasticity [147].

The mesocorticolimbic circuit is experiencing changes in synaptic strength and plasticity, especially in the ventral tegmental area (VTA) [148]. Potentiation is induced at excitatory synapses in the VTA by exposure to drugs such as cocaine, amphetamine, morphine, ethanol, nicotine, and benzodiazepines [148]. When drugs are administered non-contingently, the drug-evoked potentiation is temporary. However, when drugs are self-administered, it becomes persistent and can last up to 90 days after withdrawal [149]. The ratio of AMPA-mediated to NMDA-mediated excitatory postsynaptic currents (AMPA/NMDA ratio) is altered as a result of medication-induced synaptic modifications [148]. It is hypothesised that the development of drug-cue linkages is a consequence of “pathological” reward learning, which is initiated by an increase in synaptic strength in the VTA [148]. Considered a possible neural coding akin to long-term potentiation (LTP), the plasticity seen in VTA dopamine neurons, especially the potentiation of glutamatergic synapses, may be crucial for early drug-induced behavioural reactions. It has been demonstrated that ethanol, cocaine, and morphine impact GABA-mediated synaptic currents as well as NMDA-dependent long-term potentiation at inhibitory synapses (LTPGABA), which determines inhibitory synaptic plasticity [148]. The intricacy of neuroplasticity in addiction is further demonstrated by the modifications in inhibitory synapses, which lead to variations in the firing rate of VTA dopamine neurons [148].

Another well-known brain region that exhibits significant levels of synaptic plasticity is the hippocampus, which is frequently measured by alterations in LTP [150]. Due to the hippocampus’s great degree of plasticity and capacity to maintain declarative and contextual memories, drugs may be able to alter the hippocampal function in ways that have a significant impact on behaviour [150]. Research has demonstrated that withdrawal from methamphetamine causes impairments in hippocampal LTP, which are accompanied with deficiencies in hippocampus-dependent learning and memory [151,152]. These findings suggest that amphetamine withdrawal impairs cognition, particularly hippocampal plasticity, learning, and memory that are dependent on the hippocampal region.

Our hypothesis is that mindfulness may induce neuroplasticity at many levels of the body, which contributes to the reduction of symptoms of methamphetamine dependency. Below are some areas where neuroplasticity can occur.

### 6.1. Effect on Neurogenesis in Several Brain Structures, Primarily in the Hippocampus

The hippocampus is a key component of the network that supports memory function [153]. In the context of neurobiological changes that may help with the development of addiction-like behaviours and relapse tendencies, the hippocampus is important [154,155,156]. For instance, information regarding environmental signals connected to the drug is stored in the hippocampus [150,157,158,159]. When the person experiences those associated environmental signals, these memories aid in the development of a conditioned response or craving [159]. The fact that between 40% and 60% of addicts have at least one relapse episode following an initial period of recovery tends to lend credence to this [159]. Increasing amounts of evidence point to the importance of adult hippocampal neurogenesis in hippocampus-dependent learning and memory [160,161]. It is crucial to assess the role that adult neurogenesis plays in the addictive process and the maladaptive plasticity that methamphetamine causes in the hippocampus [159]. One way to measure neuroplasticity is through changes in brain density as measured by fMRI [162]. Methamphetamine dependency and addiction-like behaviour have been associated with an altered hippocampus morphology [163,164], decreased volume of limbic-related structures [165,166], and hippocampal-related deficiencies in learning and memory [167,168], according to evidence from clinical and preclinical research.

A neuroimaging study’s detection of increased volume in a specific structure can be explained by the development of additional neurons [169]. Mindfulness increases the volume of gray matter in many brain structures, including the hippocampus [170]. There is strong evidence that learning causes adult neurogenesis in the hippocampus [171]. Learning speeds up the dendritic trees of developing neurons and facilitates their integration into the functioning neural networks of the hippocampus [169]. Mindfulness meditation can be considered a process of learning and acquiring new skills and may promote neurogenesis in the hippocampus. The focused and non-judgmental awareness cultivated during mindfulness meditation might create an environment conducive to the formation and integration of new neurons. Adult neurogenesis is stimulated by environmental enrichment and exposure to learning cues such as mindfulness [172]. Mindfulness-induced neurogenesis may accelerate the maturation of dendritic trees in newborn neurons [173]. Dendritic maturation is crucial for the integration of these neurons into existing neural circuits, enhancing synaptic connections and network functionality [173]. The heightened attention and mental training inherent in mindfulness may facilitate this maturation process.

Additionally, mindfulness practices may contribute to improved pattern separation learning, a function associated with the hippocampus [174]. Studies in mice have shown that increasing adult hippocampal neurogenesis enhances pattern separation abilities [175]. The enhanced cognitive skills associated with mindfulness may, therefore, be linked to the formation of new neurons in the hippocampus. Mindfulness-induced neurogenic changes in the hippocampus may restore normal learning patterns, which may result in a behavioural change towards methamphetamine withdrawal.

### 6.2. Effects on Neuroinflammation

It has been demonstrated that pro-inflammatory cytokines can trigger neuronal cell death, and that neuronal cells are extremely vulnerable to pro-inflammatory cytokine-induced damage [176]. Methamphetamine is known to cause an excessive amount of DA and Glu to be released, which in turn causes neuronal inflammation [3]. According to Kohno et al. [177], the released dopamine (DA) is oxidised to generate hazardous quinones which cause presynaptic membrane damage through oxidative stress, mitochondrial malfunction, and the subsequent generation of hydrogen peroxide and peroxide radicals. The reaction to synapses and neuroinflammation is exacerbated by the disruption of mitochondrial energy metabolism and the release of inflammatory cytokines [178,179]. Targeting microglia, the innate immune cells of the central nervous system, has reportedly been linked to METH-induced neuroinflammation [180]. Toll-like receptor 4 (TLR4), which is involved in the immunological monitoring of pathogens and exogenous small chemicals, is linked to METH-mediated activation of microglia [181]. Tumour necrosis factor (TNF)-α, interleukin (IL)-1α, 1β, and IL-6 are among the inflammatory mediators that are increased when METH activates TLR4 [182]. Mindfulness has been shown to lower these markers of neuroinflammation in a variety of disorders [183,184]. Neuroinflammation negatively affects adult hippocampal neurogenesis and cognition [185]. It has been demonstrated that microglia play a significant role in adult hippocampus neurogenesis [185]. Ramified microglia are engaged in the death of newborn cells that are unable to integrate into the existing circuitry, in addition to pruning newly formed cells and providing trophic support for them when inflammation is absent [185]. It is primarily stated that the pro-inflammatory cytokine TNF-α has anti-neurogenic actions in the hippocampal regions [185]. By lowering the levels of pro-inflammatory factors, a practice such as mindfulness can increase neurogenesis in the hippocampus, contributing to improved cognitive function and craving control in people addicted to methamphetamine.

### 6.3. Neurotrophic and Vascular Effect of Mindfulness on Neuroplasticity in Methamphetamine Dependency

It is known that due to the practice of mindfulness, there are increasing tendencies in the volume of numerous brain regions and a slowing of atrophy in certain regions. The formation of new capillaries in the brain, an increase in the length and quantity of dendritic connections between neurons, and an increase in neurotrophic factors such as brain-derived neurotrophic factor [186] and insulin-like growth factor [187], that promote neurogenesis [188,189] and angiogenesis [190], may be some of the possible mechanisms by which mindfulness causes changes in the volume of the brain. In animals, the hippocampus is thought to be the primary source of BDNF [191,192]. According to reports, this neurotrophin can pass through the blood–brain barrier [193], and peripherally circulating levels of BDNF are linked to various aspects of brain function [194,195]. Peripheral BDNF concentrations are positively correlated with hippocampal size and cognitive function [196,197]. Similarly, the evidence points to the potential anti-inflammatory properties of BDNF on the brain [198,199,200]. Thus, one may speculate on how mindfulness could increase BDNF in the context of methamphetamine dependency. A potential mechanism through which mindfulness might increase BDNF levels could be related to the modulation of the BDNF-TrkB signaling pathway and its impact on the structural plasticity of dopaminergic neurons [201]. The BDNF-TrkB signaling pathway plays a crucial role in shaping the structural plasticity of dopaminergic neurons, particularly in response to addictive drugs [201]. Elevated BDNF levels may enhance the activation of TrkB receptors in dopaminergic neurons, promoting structural changes [201]. Structural plasticity, such as changes in soma size and dendritic arborisation, is associated with the long-lasting effects of addictive drugs [201]. Mindfulness-induced increases in BDNF could potentially counteract or modulate these structural changes, offering protection against addiction-related neuroadaptations. Furthermore, chronic stress has been linked to alterations in BDNF-TrkB signaling [202], providing a potential link between mindfulness, stress reduction, and the modulation of neurotrophic factors in the context of methamphetamine dependency. By reducing stress, mindfulness may contribute to maintaining or enhancing BDNF expression, particularly in dopaminergic neurons, and counteract the negative impact of addictive drugs. Secondly, mindfulness practices may enhance the overall neurotrophic signaling in the brain. This could involve increasing the availability or sensitivity of TrkB receptors, facilitating the binding of BDNF, and subsequently activating downstream signaling pathways essential for structural plasticity in dopaminergic neurons. Thirdly, mindfulness may contribute to the normalisation of cellular pathways disrupted by addictive drugs. If methamphetamine alters the balance of BDNF-TrkB signaling [203], mindfulness practices could help restore homeostasis, leading to increased BDNF levels and promoting structural plasticity in the dopaminergic neurons. The activation of these pathways by mindfulness could contribute to the prevention or reversal of the structural changes associated with addiction, potentially mitigating the reinforcing effects of drugs. Hence, through decreases in neuronal loss brought on by stress and vascular conditions, mindfulness may have an impact on hippocampus volume. Chronic methamphetamine use can cause microvascular damage, which can result in microbleeds and the gradual loss of neurons [204]. Mindfulness helps to improve cardiovascular health and reduce stress [205,206,207].

## 7. Research Limitations and Future Perspectives

Although the studies included in this review provide valuable insight into the potential of mindfulness-based techniques in the treatment of methamphetamine dependency, there are several limitations that must be considered when evaluating the results and designing future studies.

### 7.1. Heterogeneity in Study Designs

There was considerable heterogeneity among the included studies in terms of study designs, intervention protocols, participant characteristics, and outcome measures. This makes the comparison and synthesis of research results difficult. Future research should aim to use standardised methodologies to strengthen the generalisability of the findings. Consistent mindfulness session durations, frequency, and the type of mindfulness method used can facilitate clearer comparisons between subsequent studies and those already conducted. Variation in participant characteristics, including age, gender, and the severity of methamphetamine dependence (duration of dependence, duration of withdrawal, baseline craving, and other parameters), is another set of potential confounding factors. The pharmacokinetics of methamphetamine, behavioural, cognitive, and structural alterations in the brain, as well as the drug’s effects on neurotransmitter systems and molecular mechanisms, are all influenced by gender [10]. In future research, it is necessary to create samples of subjects who are homogeneous in terms of gender, age, and other parameters mentioned above. Variation in outcome measures across studies is another aspect that makes comparisons between studies difficult. The included studies used a variety of outcome measures, hence to facilitate comparison between past trials, then future studies should use the same measures as previous studies.

### 7.2. Assessment of Variables Influencing Treatment Success for Methamphetamine Dependency

The success of methamphetamine dependency treatment is influenced by a number of sociodemographic, clinical, psychological, cognitive, and physiological factors [208]. Future studies should identify these factors and examine their impact on treatment outcomes.

### 7.3. Small Sample Size

Some of the included studies had relatively small sample sizes, which limits the reliability of the results and makes it impossible to calculate the statistical power of the findings. In future studies, researchers should aim to increase participant enrollment in studies, ensuring an adequately powered sample that better represents the diversity of individuals affected by methamphetamine dependency.

### 7.4. Lack of Long-Term Follow-Up

A number of studies lack long-term follow-up data, which makes it difficult for us to evaluate the long-term benefits and resilience of mindfulness-based therapies for methamphetamine dependency. Extended follow-up assessments that monitor individuals after the initial post-intervention period should be a feature of future studies. Studies that are longitudinal and include follow-up intervals of six months, a year, or longer can offer important information about how the intervention effects are maintained and how to potentially prevent relapse. An extensive assessment of the relapse rates over a prolonged duration should be part of the long-term follow-up. Determining the long-term effectiveness of mindfulness-based interventions in preventing relapses requires an understanding of the substance use trajectory following the session. It is equally critical to investigate ways that support the continuation of mindfulness practices after the intervention period. Researching what makes people use mindfulness practices consistently can help design solutions that help people with methamphetamine dependency in the long run. A detailed picture of how the effects of mindfulness programs change over time can be obtained by directly comparing short- and long-term outcomes. This comparison can help clarify the dynamics of intervention benefits over time. Long-term follow-up evaluations ought to be a fundamental part of the intervention design, according to researchers. Making plans for long-term assessments in advance guarantees that the information required to make judgments regarding the long-term effects of mindfulness therapies is gathered.

### 7.5. Potential Bias in Self-Reported Measures

The dependence of the evaluated studies on self-reported measures raises the possibility of bias since people who are addicted to methamphetamine may underreport or give socially acceptable answers. Potential bias can be lessened by combining self-reported data with objective outcome indicators, such as behavioural observations or biological markers. Objective measurements lessen the reliance on participant self-disclosure by offering a more verifiable assessment of important variables. It is crucial to carry out validation research to evaluate the precision of self-reported metrics employed in the context of methamphetamine dependency. Assessing the validity and dependability of self-reported data can be aided by comparison with objective indicators and approved instruments. When collecting data, it can be beneficial to emphasise participant confidentiality and anonymity to promote more truthful and accurate reporting. Establishing a secure space for sharing information reduces the influence of social desirability bias and encourages honest self-evaluation. Compiling information from other sources such as ancillary reports from peers or clinicians might offer a more thorough comprehension of participants’ experiences. Triangulation is strengthened by using a variety of data sources, increasing the overall validity of study conclusions.

### 7.6. Lack of Studies Examining the Impact of Mindfulness on Neuroplasticity in the Context of Methamphetamine Dependency

Unfortunately, the studies included in the review did not use any neurophysiological or neuroimaging measures to examine the effects of mindfulness. Future studies should include fMRI neuroimaging, EEG, and measurements of various blood parameters (BDNF, pro-inflammatory factors, IGF) to confirm the hypotheses about the neuroplastic and neurogenic effects of mindfulness on methamphetamine dependency.

### 7.7. Investigating the Impact of Communication with a Mindfulness Therapist on Addiction Treatment Outcomes

Ivanova and Giannouli [209] raise an important issue in psychotherapy for the treatment of alcohol addiction. Namely, that communication between the patient and the psychologist is an important element of therapeutic interaction. The authors emphasise that when working with addicts, it is important to ensure that the patient has the feeling that the treatment will be delivered to them as a person, and that the therapeutic work will be related to the problems reported by the patient. Future research on the use of mindfulness in methamphetamine dependency should monitor and evaluate the degree/quality of communication between mindfulness therapists and methamphetamine-dependent patients. Additional important aspects of working with addicted patients, including gender differences, are described in [209].

### 7.8. Examining the Impact of Gender on the Outcome of Methamphetamine Dependency Treatment through Mindfulness

There are gender differences in the effects of treatment for methamphetamine dependency. Evidence shows that women are more responsive to treatment and benefit more from various treatment programs [210]. It is therefore expected that the situation may be similar in the case of mindfulness meditation. Future studies should control for the effect of gender on treatment outcomes and create gender-homogenous patient samples (all women vs. all men).

## 8. Conclusions

The results of the 10 studies included in the review suggest that mindfulness-based techniques are effective in alleviating the symptoms of methamphetamine dependency. Moreover, combining mindfulness with transcranial direct current stimulation is more effective than using a single intervention alone. The most positive results were shown in reducing craving. The work explains a number of psychological mechanisms that may occur in the treatment of methamphetamine dependency through mindfulness. An attempt was made to prove that mindfulness can induce neuroplastic changes (increase in the production of BDNF, increase in the volume of various brain areas), which reduce the symptoms of addiction. Despite these promising findings, there is still much to explore. Future research should ensure appropriate sample size and homogeneity, and use observation periods, including neuroimaging and neurophysiological measures, to detect mindfulness-induced neuroplastic changes.

## Figures and Tables

**Table 1:** Studies included in review.

Study	Type of Mindfulness Intervention	Participants	Number of Mindfulness Sessions	Results
[74]	Mindfulness-based relapse prevention	N = 40, 20 in the mindfulness group, 20 in the control group	Eight 90-min therapy sessions, one per week	- drug craving was alleviated, - improved emotion regulation.
[75]	Mindfulness-based therapy and counseling	N = 70, 35 in the mindfulness group, 35 in the control group	Weekly 90–120-min mindfulness practice sessions over an eight-week period	- decrease in craving levels, - significantly lower depression scores, - lower stress scores, - the rate of people experiencing a methamphetamine relapse was notably lower.
[76]	Mindfulness-based relapse prevention	N = 41, 21 in the mindfulness group, 20 in the control group	Sixteen sessions of 2-h MBRP interventions, twice a week over an eight-week period	- no effect of MBRP on craving, - no effect of MBRP on depression scores, - no effect of MBRP on anxiety scores.
[77]	Mindfulness-based cognitive therapy	N = 30, 15 in the mindfulness group, 15 in the control group	Eight 60-min sessions	- reduction of craving, - reduction of stress scores, - reduction of cortisol levels.
[78]	Mindfulness-based relapse prevention	N = 30, 15 in the mindfulness group, 15 in the control group	Eight 1.5-h sessions over a two-month period	- reduction of aggression, - reduction of craving.
[79]	Mindfulness-based substance abuse treatment	N = 40, 20 in the mindfulness group, 20 in the control group	Twelve sessions, with two sessions lasting 50–60-min per week	- reduction of risky behaviour and improved decision-making, - improved cognitive flexibility, - improved working memory, - improved response inhibition.
[80]	Mindfulness-based relapse prevention	N = 30, 15 in the mindfulness group, 15 in the control group	Nine 1-h mindfulness sessions (twice a week)	- decreased likelihood of drug use and cravings, - decreased depression score.
[81]	Mindfulness-based substance abuse treatment	N = 80, 20 in the tDCS group, 20 in the mindfulness group 20 in the combined intervention group 20 in the sham group	Twelve mindfulness sessions were held over the course of six weeks, with two sessions per week and a 72-h gap between them. Sessions were 45–50 min	- craving scores improved with the combination of MBSAT and tDCS and MBSAT alone, but the improvement was greater with the combined intervention. increase and decrease of Accuracy Go and Accuracy No-Go, respectively, after the combined tDCS + MBSAT intervention, but not after MBSAT alone, - MBSAT and the combination of tDCS and MBSAT improved accuracy and response time in the WM task, but the improvement was greater after the combined intervention, - significant decrease and increase of WCST persistent errors and completed categories after MBSAT and the combination of tDCS and MBSAT, but the improvement was greater after the combined intervention, - significant decrease of BART Adjusted value and Max pumping, respectively, after MBSAT and combined tDCS and MBSAT, but the improvement was greater after the combined intervention.
[82]	Mindfulness-based substance abuse treatment	N = 48, 15 in the mindfulness group, 17 in the combined intervention group, 16 in the sham group	Twelve mindfulness sessions were held over the course of six weeks, with two sessions per week and a 72-h gap between them. Sessions were 45–50 minn	- craving scores improved with the combination of MBSAT and tDCS and MBSAT alone, but the improvement was greater with the combined intervention, - improvement in AB after MBSAT and the combination of tDCS and MBSAT, but after the combined intervention the improvement was greater
[83]	Mindfulness-based substance abuse treatment	N = 80, 20 in the tDCS group, 20 in the mindfulness group 20 in the combined intervention group 20 in the sham group	The 12-session mindfulness regimen (two sessions per week) was followed by the MBSAT group. The duration of each mindfulness treatment session was 40–50 min	- craving scores improved with the combination of MBSAT and tDCS and MBSAT alone, but the improvement was greater with the combined intervention. - both MBSAT alone and the combination of tDCS and MBSAT improved anxiety and stress scores, while the improvement was greater after the combined intervention, - both MBSAT alone and the combination of tDCS and MBSAT improved depression scores, while the improvement was greater after combined intervention.

## Data Availability

No new data were created or analysed in this study. Data sharing is not applicable to this article.
